# Thyroid Hormones Accelerate Initiation of Skeletogenesis via MAPK (ERK1/2) in Larval Sea Urchins (*Strongylocentrotus purpuratus*)

**DOI:** 10.3389/fendo.2018.00439

**Published:** 2018-08-06

**Authors:** Elias Taylor, Andreas Heyland

**Affiliations:** Department of Integrative Biology, University of Guelph, Guelph, ON, Canada

**Keywords:** thyroid hormones, thyroid hormone receptor, MAPK, ERK1/2, integrin, non-genomic, sea urchin, skeletogenesis

## Abstract

Thyroid hormones are important regulators of development and metabolism in animals. Their function via genomic and non-genomic actions is well-established in vertebrate species but remains largely elusive among invertebrates. Previous work suggests that thyroid hormones, principally 3,5,3′,5′-Tetraiodo-L-thyronine (T4), regulate development to metamorphosis in sea urchins. Here we show that thyroid hormones, including T4, 3,5,3′-triiodo-l-thyronine (T3), and 3,5-Diiodothyronine (T2) accelerate initiation of skeletogenesis in sea urchin gastrulae and pluteus larvae of the sea urchin *Strongylocentrotus purpuratus*, as measured by skeletal spicule formation. Fluorescently conjugated hormones show T4 binding to primary mesenchyme cells in sea urchin gastrulae. Furthermore, our investigation of TH mediated skeletogenesis shows that Ets1, a transcription factor controlling initiation of skeletogenesis, is a target of activated (phosphorylated) mitogen-activated protein kinase [MAPK; extracellular signal-regulated kinase 1/2 (ERK1/2)]. As well, we show that PD98059, an inhibitor of ERK1/2 MAPK signaling, prevents the T4 mediated acceleration of skeletogenesis and upregulation of Ets1. In contrast, SB203580, an inhibitor of p38 MAPK signaling, did not inhibit the effect of T4. Immunohistochemistry revealed that T4 causes phosphorylation of ERK1/2 in presumptive primary mesenchyme cells and the basal membrane of epithelial cells in the gastrula. Pre-incubation of sea urchin gastrulae with RGD peptide, a competitive inhibitor of TH binding to integrins, inhibited the effect of T4 on skeletogenesis. Together, these experiments provide evidence that T4 acts via a MAPK- (ERK1/2) mediated integrin membrane receptor to accelerate skeletogenesis in sea urchin mesenchyme cells. These findings shed light, for the first time, on a putative non-genomic pathway of TH action in a non-chordate deuterostome and help elucidate the evolutionary history of TH signaling in animals.

## Introduction

**Figure 1:**
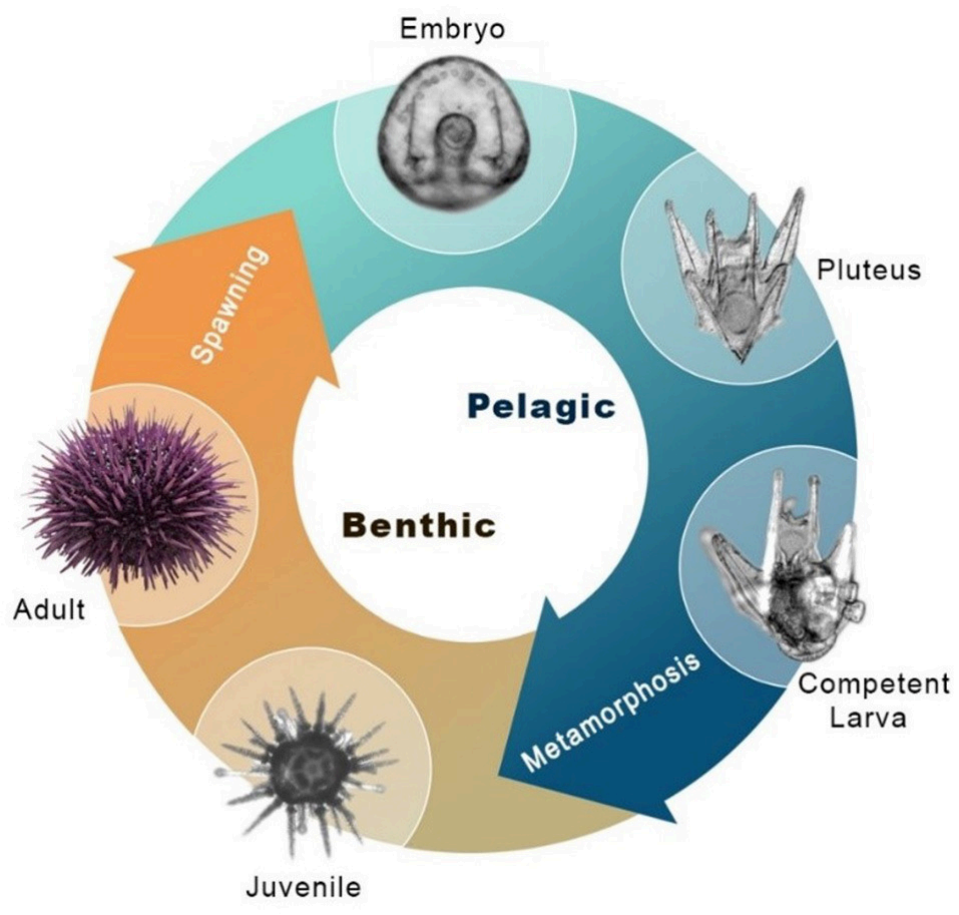
Sea urchin indirect life history. Major stages of the indirect life cycle of *S. purpuratus* during which skeletogenesis occurs. Post-hatching, the embryo grows larval arms, becoming a free-swimming pluteus larva. The pluteus feeds on plankton in the water column, accumulating resources for metamorphosis. Prior to attaining metamorphic competence, the larva grows a juvenile rudiment with skeletal structures necessary for survival as a juvenile, including adult spines and tube feet. Also pictured is the metamorphosed juvenile, and the adult sea urchin.

**Figure 2:**
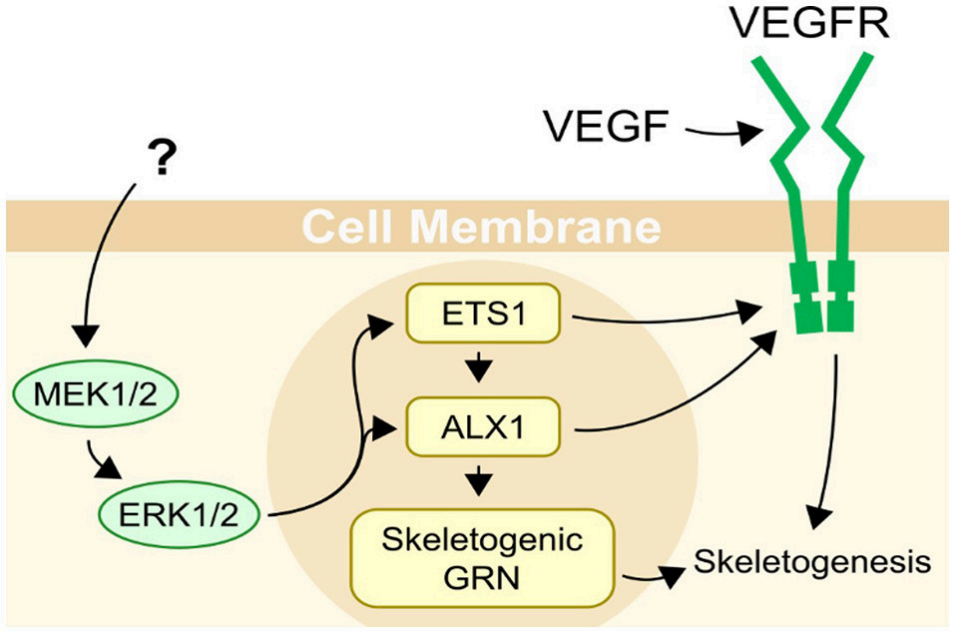
Regulation of skeletogenesis in sea urchins. Two main inputs are known to be necessary for skeletogenesis in sea urchins, VEGF secreted by ectodermal cells, and a MAPK (ERK1/2) cascade with an unknown trigger. ETS1 and ALX1 are transcription factors, and are important regulators of skeletogenesis in sea urchins, controlling almost half of the genes differentially expressed in primary mesenchyme cells (11). Their activity is necessary for skeletogenesis to occur. Both Ets1 and Alx1 are activated or upregulated by MAPK (ERK1/2).

Echinoderms are the closest relatives to the vertebrates that produce a mineralized skeleton, though they independently evolved the capability for biomineralization (1). Unique among echinoderms, sea urchins produce an extensive skeleton during both major life cycle phases: the benthic adult and the planktonic pluteus larva (Figure 1). In the larva, the skeleton supports the larval arms, structures essential for feeding and locomotion (2, 3). The skeletal structures are integral to the sea urchin pluteus larva, which feeds in the plankton and also develops juvenile structures within the echinus rudiment in preparation for metamorphosis (4). During settlement, the rudiment is everted from the larva and while the juvenile begins its life in the benthic habitat, the larval structures are resorbed, likely involving programmed cell death under the control of histamine (5). Larval skeletogenesis (the process by which skeleton is formed *de novo*) appears to be regulated by a gene regulatory module that is also responsible for juvenile and adult skeletogenesis (6). This gene regulatory mechanism has been the focus of extensive research in the purple sea urchin *Strongylocentrotus purpuratus* and related species [for example, (7–11)]. Specifically, a MAPK cascade phosphorylating the transcription factors Ets1 and Alx1 is necessary for skeletogenesis [Figure 2; (12)].

Previous research has shown that vertebrate thyroid hormones (THs), specifically 3,5,3′,5′-tetraiodo-L-thyronine (T4) change the developmental trajectory of echinoid larvae toward settlement, by accelerating the development of juvenile structures in the rudiment (13–15). In contrast to the accelerated development of juvenile skeletons, larval arms are reduced when treated with THs (T4 and T3), a phenotype suggesting differential regulation of skeletogenesis in the larval vs. juvenile developmental program. For example, irregular urchins (sand dollars) exhibit accelerated development in response to T4, and delayed development in response to inhibitors of TH synthesis (14–16). Furthermore, TH levels in sea urchin larvae rise throughout development, peaking at metamorphic competence [(16, 17), possible origins discussed in (18)].

Due to the striking evidence for TH effects on larval and juvenile skeletons in sea urchins, we hypothesized that THs can regulate this process and we set out to analyze the mechanism through which the gene regulatory network underlying skeletogenesis is activated by TH. MAPK signaling is known to be necessary for skeletogenesis in sea urchins and is activated by TH binding to an integrin during vertebrate angiogenesis. Due to these similarities, we hypothesized and tested a comparable role of integrin-mediated MAPK signaling in the sea urchin response to THs.

## Materials and methods

### Adult urchins

Adult urchins (*S. purpuratus*) were shipped from Monterey, CA, where they were collected by diving and subsequently kept in tanks of artificial seawater at the Hagen Aqualab (University of Guelph, ON). The adults were fed a diet of kelp (*Macrocystis pyrifera*, and *Kombu* spp.) every 2–3 days. Temperature was maintained at 12–14°C and salinity at 31 ppt).

### Culturing of embryos and larvae

Urchins were spawned by injecting 0.5–2 mL of 0.5 M KCl, depending on the size of the urchin. Sperm was collected dry by pipetting it directly from the gonopores. Females were inverted over a beaker of filtered artificial seawater (0.2 μm–FASW) to collect eggs. After spawning, eggs were gently passed through a 120 μM Nitex™ mesh to remove debris, before being washed twice with FASW. Diluted sperm (approx. 1:100) was titrated into the beaker of eggs until fertilization success reached at least 90%. Fertilized eggs were washed once more with FASW to remove excess sperm and allowed to develop at 12°C in a 1 L beaker until hatching. After 48 h at 12°C, hatched embryos were transferred to 2 L beakers at a density of 1 larva/mL. Sea urchin larval cultures were maintained at 12–14°C with salinity at 31–33 g/L. Cultures were stirred constantly using a paddle system [as described in (19)] and kept on a 12:12 light cycle. The cultures were cleaned manually and had the water replaced three times weekly. At the same time, cultures were fed *Rhodomonas* sp. at a density of 6,000 cells/mL. Gastrulae were collected after 24–48 h at 12°C and staged for each experiment as indicated in the sections below. For replicate experimental treatments, these gastrulae were drawn at random from the same culture. Larval sea urchins were collected after 12–24 days at 12°C, as indicated in the sections below.

### Assay for skeletogenesis

We developed an experimental assay to characterize the effects of thyroid hormones on the rate of initiation of skeletogenesis. This assay allows for efficient scoring of initiation of skeletogenesis. We scored the presence or absence of skeletal spicules in the blastocoel of gastrulae (typically from 36 to 48 h post fertilization), or in the posterodorsal arms of pluteus larvae and calculated the proportion of individuals with skeletons. Skeletal structures were detected by polarized light microscopy on a Nikon TI Eclipse compound inverted microscope.

Larvae were kept in 24 well plates at a density of 20 embryos/mL for gastrulae, or 1 larvae/mL for pluteus larvae. The plates were kept in an incubator at 12°C on a shaker table. Water was changed and larvae were fed 6,000 c/mL *Rhodomonas* sp. every 2 days.

### Thyroid hormone exposure

The effects of thyroid hormones (T4, T3, and T2) on rate of skeletogenesis were observed at two concentrations, 1 nM and 100 nM. Triac and Tetrac, deaminated forms of T3 and T4 respectively, which can inhibit TH activity in vertebrates, were also tested. Thyroid hormones, including T4, T3, rT3, T2, Tetrac, and Triac were dissolved in DMSO at 100 mM before being diluted to 1 mM in FASW as a stock solution. Aliquoted hormones were stored at −20°C for up to 12 months. To expose larvae, THs were diluted from the stock solution by 1:10 serial dilution, to the required concentration in FASW, and larvae were placed in that FASW. FASW with DMSO and/or FASW with rT3 and DMSO was used as a vehicle control in every experiment.

Acute exposure experiments were performed using FASW, rT3, and T4, to determine the likely timescale on which thyroid hormones act on skeletogenesis. Larvae were exposed to T4 at 10 nM or 1 μM for 1 h, washed twice with FASW, and then placed in FASW for 72 h. A second group was exposed to T4 at the same concentrations for 72 h. The proportion of larvae which underwent skeletogenesis was then compared.

### MAPK inhibition

Inhibitors of MAPK phosphorylation were used to determine if TH acts via a MAPK signaling cascade, and if so, which MAPK is responsible. Embryos were pre-exposed to SB203580 (Sigma-Aldrich S8307), an inhibitor of the p38 MAPK signaling, or to PD98059 (Sigma-Aldrich P215), an inhibitor of ERK1/2 MAPK signaling at 24 h post fertilization for 1 h. They were then exposed to T4 for 20 h, after which the rate of skeletogenesis was assayed over 5 h.

### Integrin inhibition with RGD peptide

RGD peptide (Sigma-Aldrich A8052), a competitive inhibitor of the RGD binding pocket in integrin αVβ3 was used to determine whether T4 might bind to an integrin in sea urchins. Embryos were pre-exposed to RGD peptide at 24 h post fertilization for 1 h. They were then exposed to T4 for 20 h, after which the rate of skeletogenesis was assayed over 5 h.

### Immunohistochemistry of phosphorylated MAPK and PMC membranes

M8159 (mouse monoclonal, Sigma-Aldrich M8159), an antibody for phosphorylated ERK1/2, was used to quantify MAPK (ERK1/2) phosphorylation after either T4 exposure. Embryos were exposed to 100 nM T4 for 90 min prior to fixation.

In a second experiment to localize TH binding, gastrulae were also stained with a custom 6a9 antibody (mouse monoclonal, kindly provided by the Ettensohn lab at Carnegie Mellon University) for primary mesenchyme cell membranes. This was performed in conjunction with the procedure for fluorescently labeled THs described below.

Embryos were fixed for whole mount immunohistochemistry in methanol at −20°C for 5 min before being transferred to PBS. After immunohistochemistry, larvae were imaged under fluorescent light with a Cy5 filter (Nikon Bandpass filter cube, excitation 620 nm, emission 700 nm). MAPK phosphorylation levels were quantified as mean fluorescence intensity of the embryo.

### Fluorescently labeled THS

Thyroid hormones, including T4, T3, and rT3 were conjugated with rhodamine to produce fluorescently labeled thyroid hormones. Synthesis was carried out as in Cheng et al. (20), with several exceptions. Thyroid hormones were mixed with equimolar TRITC (mixed isomers, Sigma-Aldrich 95197-95-8) to conjugate rhodamine to the amino group of the thyroid hormones. Reaction was stirred and allowed to proceed for 1 h at room temperature. Products were purified by column chromatography and checked by thin layer chromatography (solvent used for both chromatographies was ethyl acetate:methanol:water, 5:2:3 volume, as in original protocol). Solvent was then evaporated, leaving behind purified rhodamine conjugated hormones. Rhodamine-reverse thyronine (RH-rT3) was synthesized by substituting rT3 for T3 in the reagents.

Live gastrulae were exposed to rhodamine-thyroxine (RH-T4), rhodamine-thyronine (RH-T3) or rhodamine-reverse thyronine (RH-rT3) at the biologically relevant concentration of 10^−8^ M. They were incubated for 30 min. Gastrulae could then be fixed for immunohistochemistry, or imaged live. Gastrulae were imaged under 540 nm light, the excitation wavelength of TRITC. Filters were used to examine only the emission wavelength of TRITC (Nikon Bandpass filter cube, excitation 540 nm, emission 605 nm).

### QRT-PCR

Quantitative real-time PCR was performed to determine the effect of T4 exposure on expression of Ets, a transcription factor regulating skeletogenesis in sea urchin embryos. Embryos were pre-exposed to PD98059, an inhibitor of ERK1/2 MAPK signaling at 24 h post fertilization for 1 h. They were then exposed to T4 for 20 h before being collected by centrifugation.

The expression of various skeletogenesis-related genes was also determined via qRT-PCR in late-stage larvae. Levels of Ets, Alx, VegfR, Sm50, and THR were measured before and after a 24 h exposure to 100 nM T4 in 24 day-old larvae which had just begun rudiment formation. Larvae were collected by pipette.

Gastrulae and larvae were lysed in TRIzol reagent at −20°C overnight. RNA extraction was carried out using a Direct-zol™ RNA MiniPrep kit, following the manufacturer's instructions (Zymo Research). Concentration and RNA purity were checked using Nanodrop 8000 Spectrophotometer (Thermo Scientific). RNA was copied to cDNA with an Applied Biosystems cDNA synthesis kit as per the provided protocol and stored at −20°C until use.

qRT-PCR was performed on a StepOne Plus with SYBR Green dye. Primer concentrations were optimized using a test mix of cDNA from 2 day, 12 day, and 25 day-old larvae. A list of primers ordered from Invitrogen, and their concentrations can be found in Table 1. Transcript levels were compared to Ubq (Ubiquitin, NM_214533) levels as a reference, using the ΔΔCt method [ΔΔCt reviewed in (22)] with primer concentrations of 300 nM. In all cases, unexposed larvae from the same culture were used as a control.

**Table 1:** Primers used in qPCR.

**Gene name**	**Forward sequence**	**Reverse sequence**	**Forward conc. (nM)**	**Reverse conc. (nM)**
Alx	CACCCGTAGAGGGCGCTATA	TGCTGGAGTCTTGCGATTCG	200	400
Ets	CGCAAAAACAAACCCAAGAT	TCTGCAGGTCACAGACGAAG	400	400
Sm50	AACAACCAGGTATGGGTGGA	TATTCGGGTTATTCGGTTGC	400	200
THR	TTCGAAGGACGATTCAGAAGA	TAACGGCATTGCTGACATTG	400	400
Ubq	CACAGGCAAGACCATCACAC	GAGAGAGTGCGACCATCCTC	300	300
VegfR	GTAAGGCCCAAAATGGAGAA	CCGAGACCTCACAGCTTACA	200	100

*All primers ordered from invitrogen*.

### Statistical analysis

In cases where the dependent variable was binary and there was a single independent variable, a Chi Square test was used to determine significant differences between groups, with Bonferroni corrected *p*-values if multiple *post-hoc* comparisons were made (*p* < 0.05 assumed to be significant). The statistic used was χ^2^ (Chi Square).

In cases where the dependent variable was binary and there were multiple independent variables, a generalized linear model (binary logistic regression) was used, with Bonferroni corrected pairwise *post-hoc* comparisons. Significance of the individual treatment groups relative to the control was tested, as well as whether treatment groups had a significant effect on the model, and whether the interaction between treatments was significant. Test statistics are reported as Wn (Wald Chi-Square). Unless otherwise noted, proportions and means are reported as ± the standard error.

## Results

### Thyroid hormones accelerate skeletogenesis

**Figure 3:**
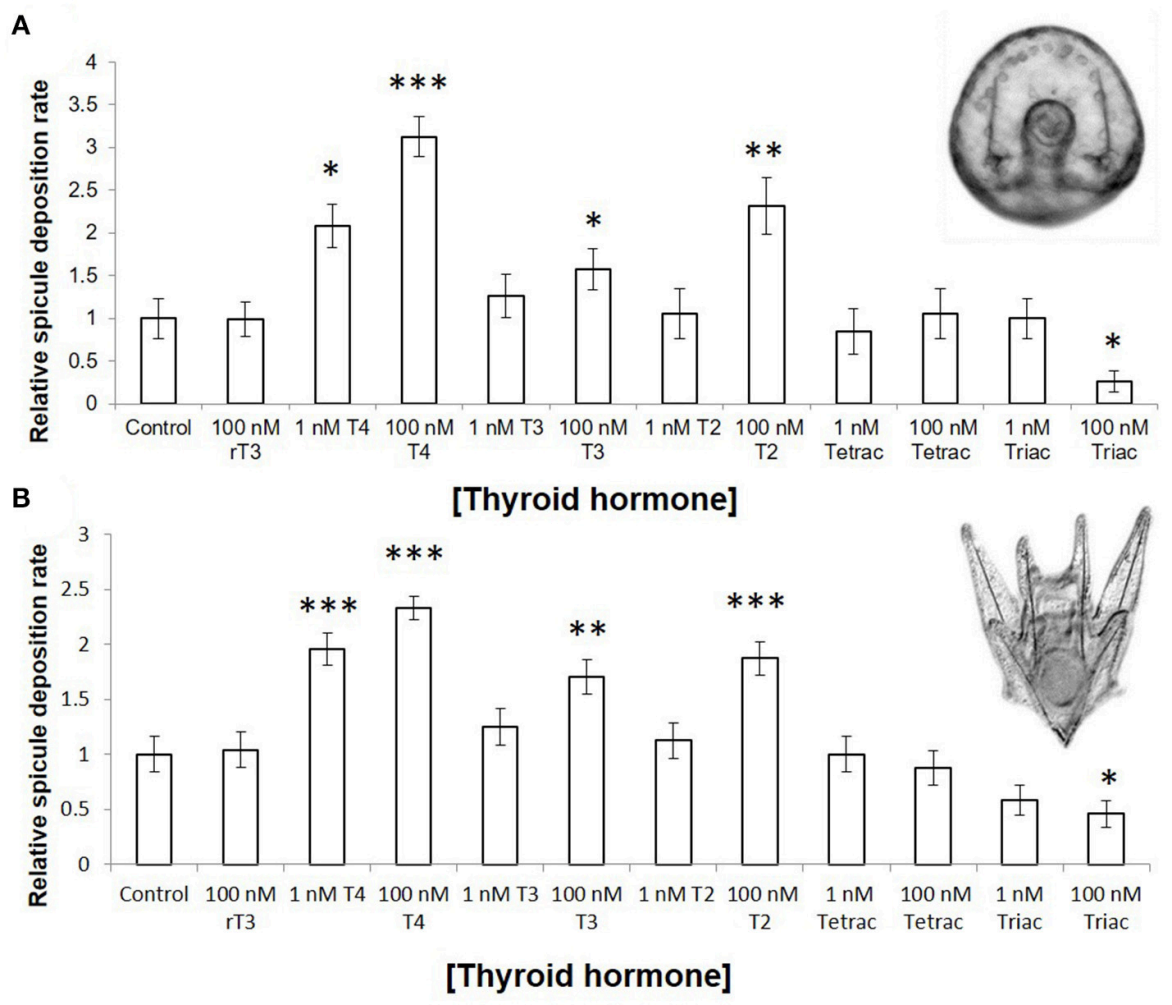
Thyroid hormones accelerate rate of skeletogenesis in gastrulae and pluteus larvae. **(A)** Gastrulae were exposed for 20 h, from 24 to 44 h post fertilization (*n* = 40–81). **(B)** Six armed larvae were exposed for 4 days, from 10 days post fertilization to 14 days post fertilization (*n* = 63). Larvae were scored at regular intervals for the presence or absence of skeletal spicules; either hourly (gastrulae) or daily (six armed larvae). Spicule deposition rate was normalized to the control. In both stages examined, T4, T3, and T2 accelerated skeletogenesis (*p*= <0.001–0.002) in a dose-dependent fashion (*p* = 0.001–0.045), while Triac inhibited skeletogenesis (*p* = 0.008). (Binary logistic regression with Bonferroni corrected *p*-values. ^*^p < 0.05, ^**^p < 0.01, ^***^p < 0.001, indicates significant difference from control group).

**Figure 4:**
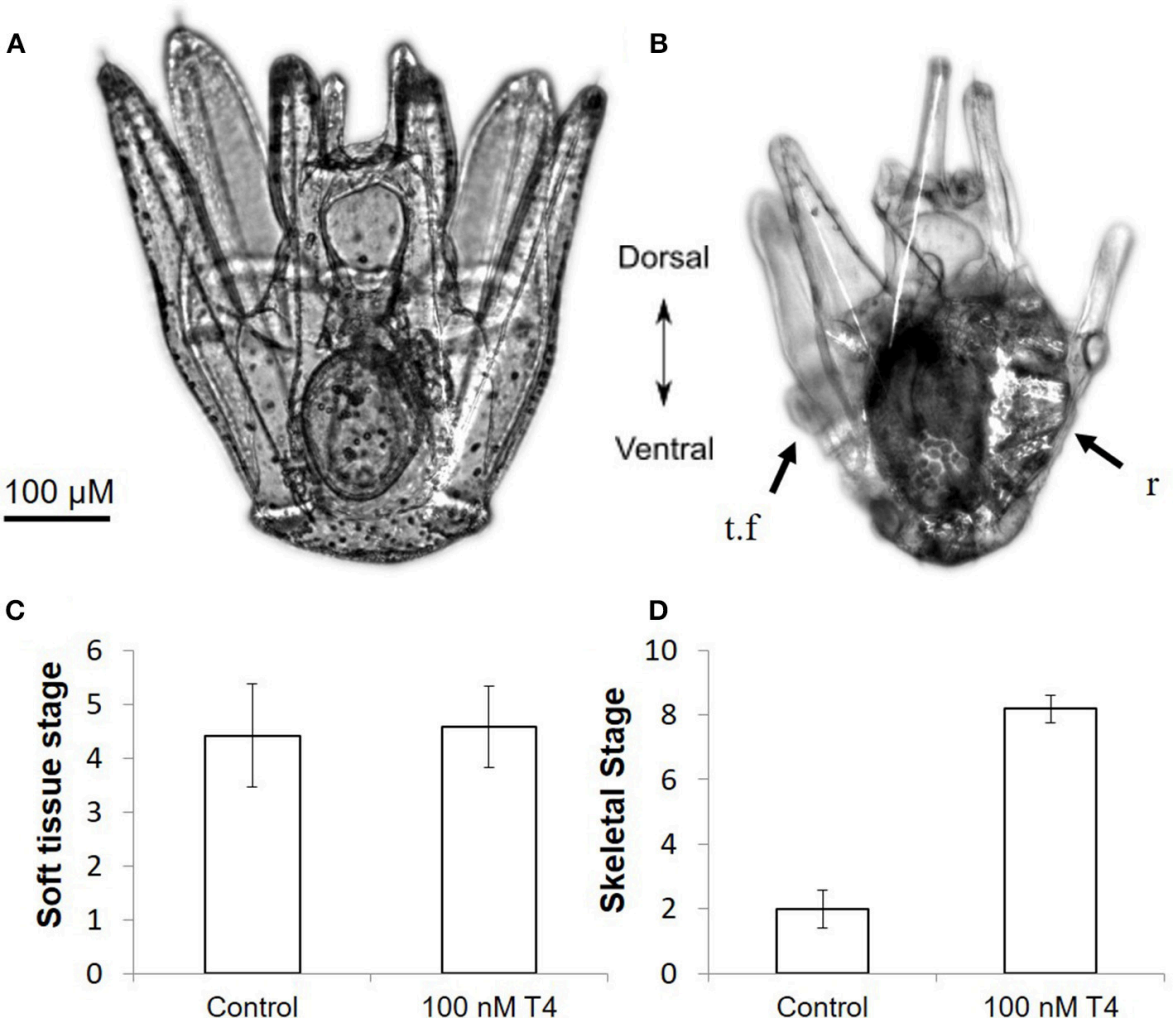
Thyroxine accelerates rudiment development in *S. purpuratus* larvae. Pictured are late stage larvae kept either without T4 **(A)** or with 100 nM T4 for 5 days **(B)**. T4 exposed larvae have significantly more developed skeletal elements in the rudiment, as well as shortened larval arms. Larvae at soft tissue stage 1 **(C)** or skeletal stage 0 **(D)** were exposed to T4 for 5 days [*n* = 12, for staging scheme, see (21)]. T4 drastically accelerated skeletogenesis in the rudiment (Mann–Whitney, *p* < 0.001) as well as accelerating other markers of metamorphic competence, including arm retraction and tube feet protrusion, but did not accelerate early soft tissue development (Mann–Whitney, *p* = 0.63). This suggests that the rudiment of late stage larvae may become responsive to T4 only as skeletal development begins. r, rudiment; t.f, tube foot.

T4, accelerates skeletogenesis in a dose dependent manner in all developmental stages tested (gastrulae, early stage, and late stage larvae–Figure 3A: gastrulae and early stage larvae; Figure 3B: late stage larvae). T4 also increased the rate of spicule formation in gastrulae and early pluteus larvae relative to both the control, and to the biologically inactive rT3 (Figure 3B; 3.1-fold in gastrulae, Wn = 48.7, *p* < 0.001; 2.3-fold in early stage larvae, Wn = 35.7, *p* < 0.001). In late stage larvae, the rudiment reached an average skeletal stage of 8.18, with growing juvenile spines and tube feet, as compared to the control with an average stage of 2.00: initial spicule formation (Figure 4, Mann–Whitney *U* = 99.0, *p* < 0.001). T4 did not significantly affect soft tissue development in the rudiment (Figure 4, Mann–Whitney *U* = 81, *p* = 0.63). High levels of T3 and T2 (100 nM, Figure 3, Wn = 32.5, 26.3, *p* < 0.001) were also found to accelerate spicule formation at the onset of skeletogenesis. Tetrac did not have an effect on skeletogenesis (Figure 3, Wn = 0.4, *p* = 0.5) while Triac inhibited skeletogenesis in gastrulae and early pluteus stages (Figure 3, Wn = 4.1, *p* < 0.05).

**Figure 5:**
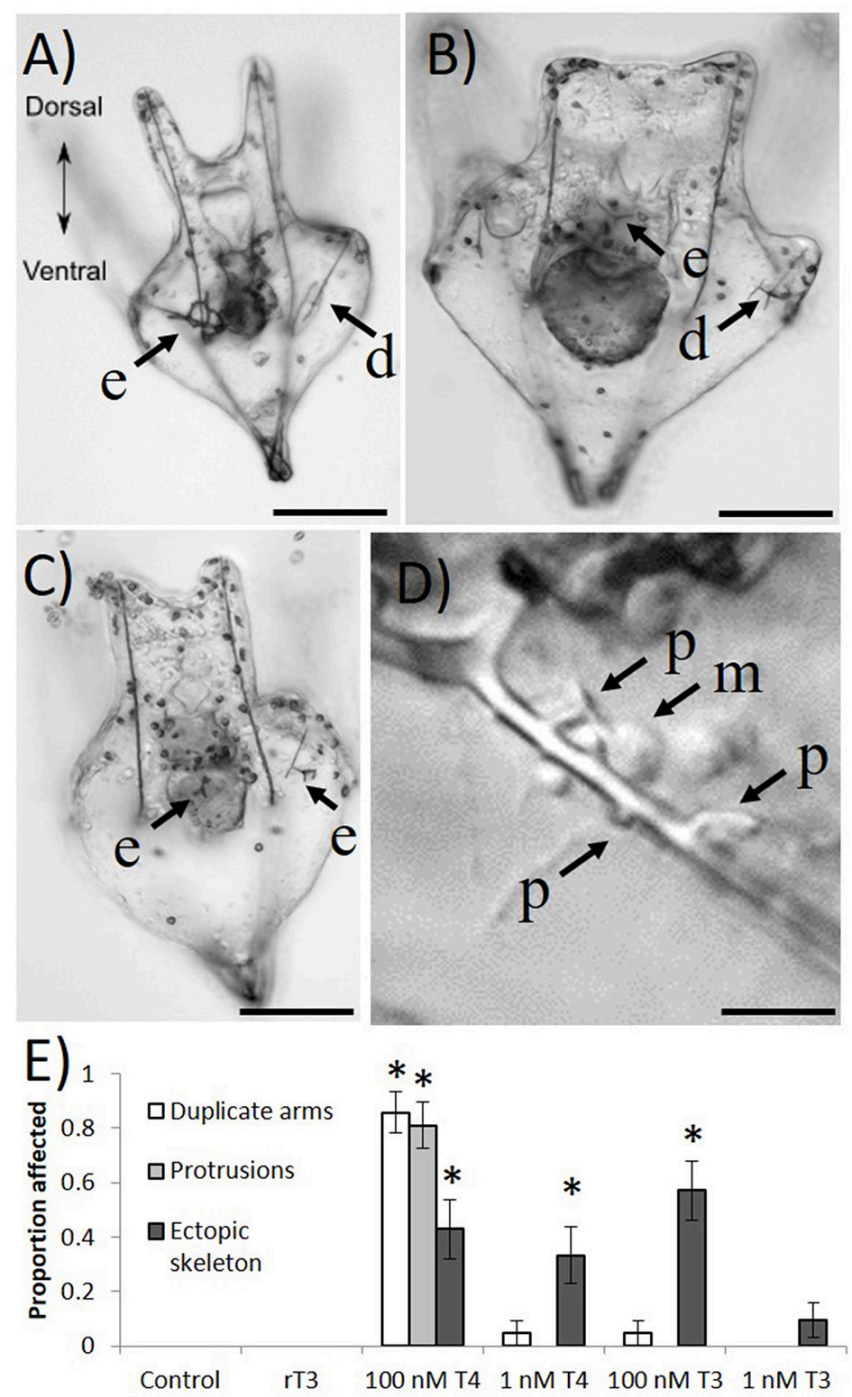
Four days exposure to thyroxine (100 nM) causes ectopic skeleton. Frequently, after exposure to high levels of THs, larvae develop skeletal abnormalities. **(A)** Skeletal rings, as well as a duplicate posterodorsal arm can be seen. **(B)** An ectopic spicule, as well as abnormal branching from the posterodorsal arm. **(C)** Several ectopic spicules. **(D)** A number of unusual skeletal protrusions on the post-oral arm, as well as presumptive primary mesenchyme cells in the process of laying down skeleton. **(E)** Only T4 and T3 caused skeletal abnormalities, with protrusions and duplicate posterodorsal arms being observed with higher exposure to T4 (*Z*-test, *p* < 0.001), where nearly every larvae examined had a skeletal aberration. Ectopic skeleton was significantly more present in all levels of T4 exposure and the higher level of T3 exposure (*Z*-test, *p* = < 0.001–0.004). No skeletal abnormalities were observed in the control or rT3 groups. d, duplicate posterodorsal arm; e, ectopic skeleton; p, skeletal protrusion; m, primary mesenchyme cell. Images were taken with differential interference contrast microscopy (DIC). Scale bars **(A–C)** (100 μm); **(D)** (20 μm). (^*^p < 0.05).

In 12 day-old larvae, we found that 1 nM T4 and 100 nM T3 can cause abnormalities in skeletal growth (Figure 5). Specifically, ectopic skeleton (Figures 5A,C; spicules forming outside the posterodorsal arms) was observed, as well as the formation of duplicate posterodorsal arms (Figures 5A,B), and skeletal protrusions along the postoral arms (Figure 5D). No specific location could be identified for ectopic skeleton formation, which occurred in a variety of positions. Only T4 and T3 caused skeletal abnormalities, with protrusions and duplicate posterodorsal arms being observed with higher frequency in the T4 treatment (Chi Square, χ^2^ = 132.9, 124.1 Bonferroni corrected *p* < 0.001), where nearly every larva examined had a skeletal aberration. Ectopic skeleton was seen at all levels of T4 exposure and the higher level of T3 exposure (Chi Square, χ^2^ = 55.6, Bonferroni corrected *p* < 0.001). No skeletal abnormalities were observed in the control or rT3 groups.

### Acute and chronic exposures to T4 show similar effects

**Figure 6:**
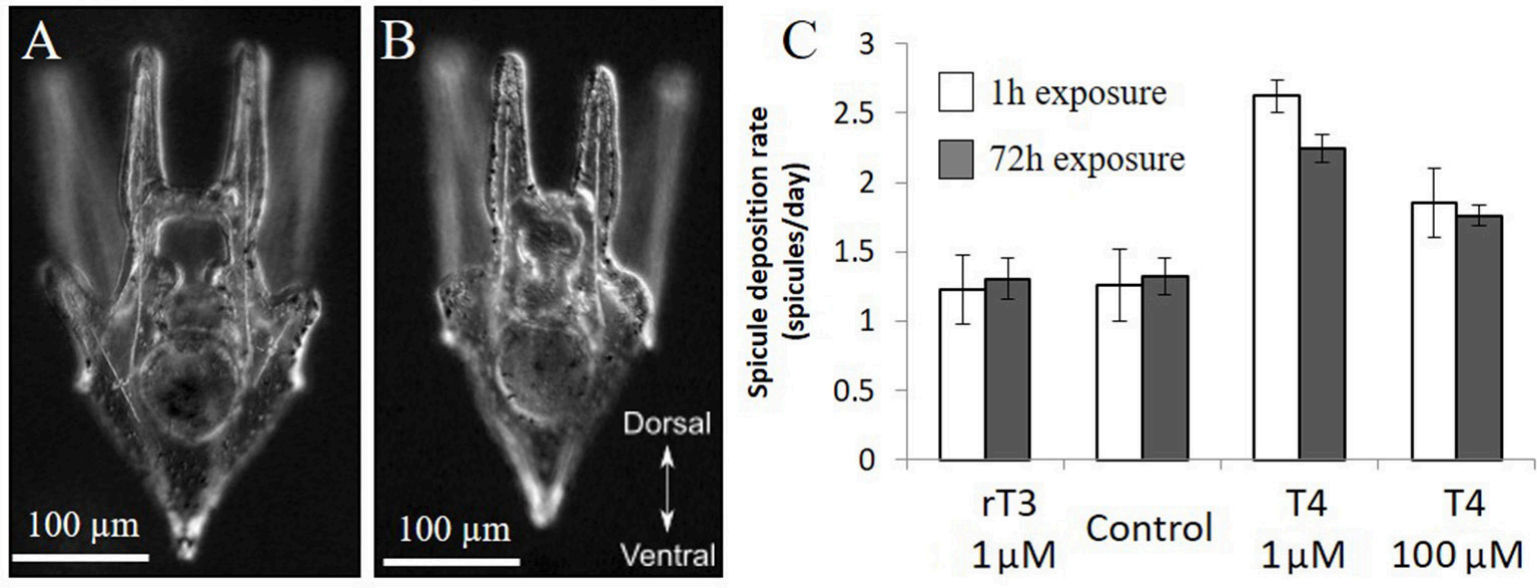
Acute and chronic exposure to T4 have similar effects. In the acute exposure groups, 12 day old larvae were exposed to rT3 or T4 for 1 h before being washed thoroughly and placed in clean seawater (*n* = 21). The chronic exposure groups were exposed for 3 days (*n* = 82). Larvae from both groups were imaged daily, and the presence or absence of spicules in the developing posterodorsal arms was noted. **(C)** Acute exposure (1 h) to thyroxine has similar effects to chronic exposure over 4 days, with high levels of T4 causing a significant increase in spicule initiation in both groups (*Z*-test, *p* < 0.01). **(A)** representative larva from T4 treatment, **(B)** representative larvae from control and rT3 treatments.

Larvae exposed to T4 for 1 h and monitored for 5 days showed a similar rate of acceleration of skeletogenesis when compared to larvae chronically exposed for 5 days. The higher concentration of T4 tested (1 nM T4) showed a significant acceleration of skeletogenesis relative to the control (Figure 6), with no discernable differences between acute and chronic exposures (Figure 6C, acute 2.6-fold, chronic 2.2-fold relative to control, χ^2^ = 15.9, *p* < 0.01). The lower concentration acute exposure (1 μM T4) did not result in a statistically significant difference in the time of onset of skeletogenesis compared to the control, although showing a trend toward accelerated skeletogenesis (χ^2^ = 2.7, *p* = 0.10).

### Inhibitors of ERK 1/2 reduce T4 effect in skeletogenesis

**Figure 7:**
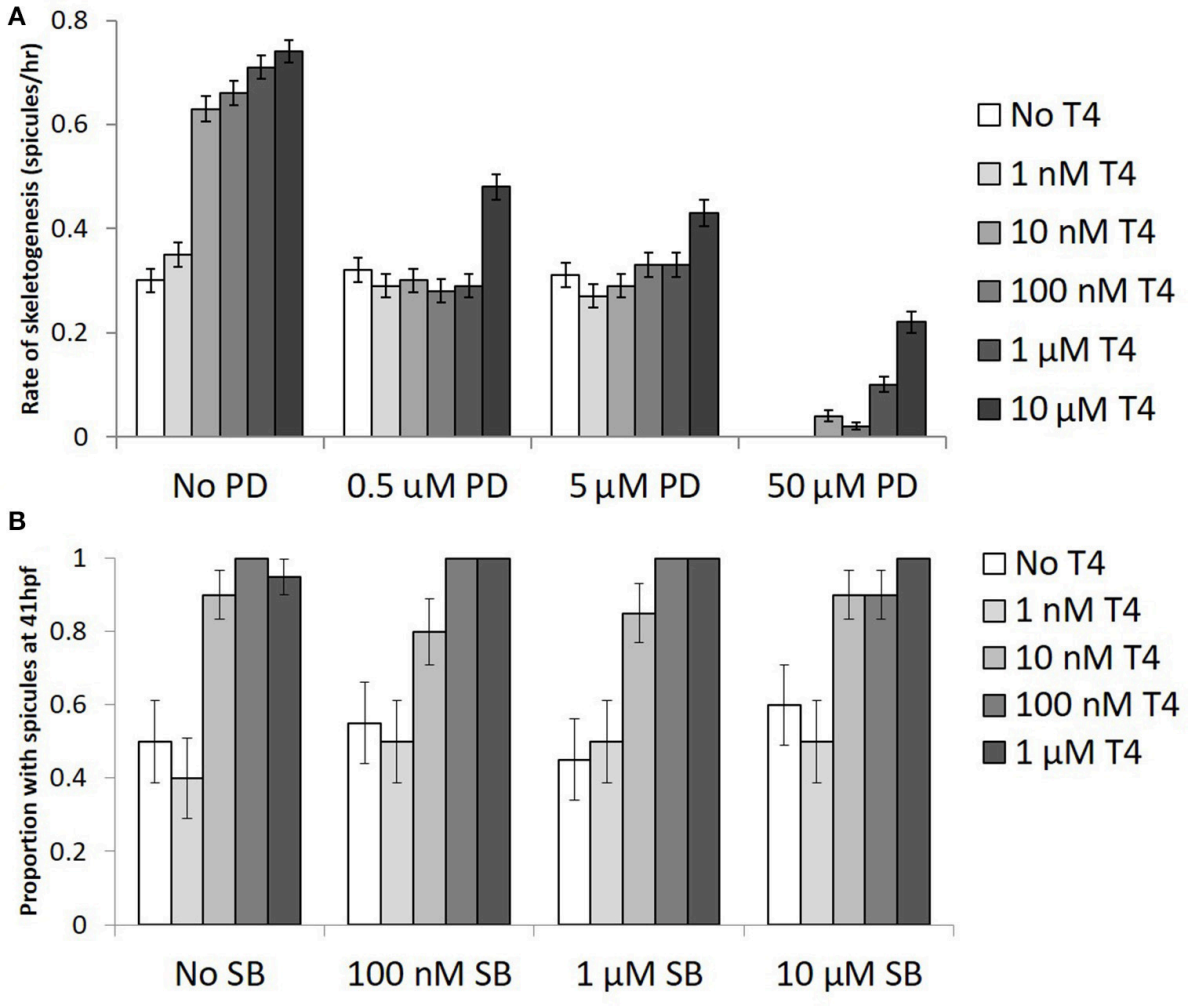
The effect of T4 on skeletogenesis is inhibited by PD98059, an ERK1/2 inhibitor but not p38 inhibitor. **(A)** Gastrulae were pre-exposed to PD98059 before being exposed to T4 for 20 h (*n* = 100). Following exposure, spicule initiation was monitored hourly for 5 h. The hypothesis that PD98059 inhibits the effect of T4 on skeletogenesis was tested using a binary logistic regression (*D* = 232, *df* = 110). T4 increased the rate of skeletogenesis, while PD98059 inhibited the effect of T4 on skeletogenesis (Bonferroni corrected *p* <0.0001). The highest levels of PD prevented skeletogenesis completely, an effect which was rescued by T4 (Bonferroni corrected *p* = 0.0012). This suggests that T4 acts through a MAPK (ERK1/2) cascade. **(B)** Gastrulae were pre-exposed to SB203580 before being exposed to T4 for 20 h (*n* = 20). Following exposure, spicule proportion at 41 hpf was observed. The hypothesis that SB inhibits skeletogenesis was tested using a binary logistic regression (*D* = 11, *df* = 12). T4 increased the rate of skeletogenesis (Bonferonni corrected *p* < 0.001), while SB had neither a significant effect on skeletogenesis nor any interaction with the effect of T4 (*p* = 0.996).

When gastrulae were exposed to two inhibitors of MAPK signaling, SB203580, and PD98059, both with and without added T4, we found that T4 induced skeletogenesis was inhibited by PD98059, but not by SB203580 (Figure 7). All concentrations of PD98059, an inhibitor of ERK1/2, significantly reduced the effect of T4 on skeletogenesis (Figure 7A). PD98059 at concentrations of 0.5 μM and 5 μM resulted in a diminished effect of T4, such that it was not statistically different from the control (binary logistic regression, Wn = 0.00, *p* = 1.00). The highest level of T4, at 10 μM, accelerated skeletogenesis even in the presence of PD98059 at concentrations up to 5 μM (Wn = 86.8, *p* < 0.001). The highest level of PD98059 tested (50 μM) completely inhibited skeletogenesis (Wn = 153.9, *p* < 0.001), with skeletogenesis being partially restored by even low levels of T4 (10 nM T4; Wn = 13.8, *p* < 0.001). The interaction between T4 and PD98059 treatment was significant (Wn = 121.5, *p* < 0.001).

SB203580, an inhibitor of p38, did not alter the effect of T4 on skeletogenesis (Figure 7B; binary logistic regression, Wn = 0.07, *p* = 1.00) at any of the three concentrations tested (100 nM, 1 μM, 10 μM; Wn = 0.01–0.06, *p* = 0.81–0.94). The interaction between SB203580 and T4 was also not significant at any concentration tested (*p* = 0.07–1.00).

### PCR of Ets1 shows upregulation in response to T4 in gastrulae

**Figure 8:**
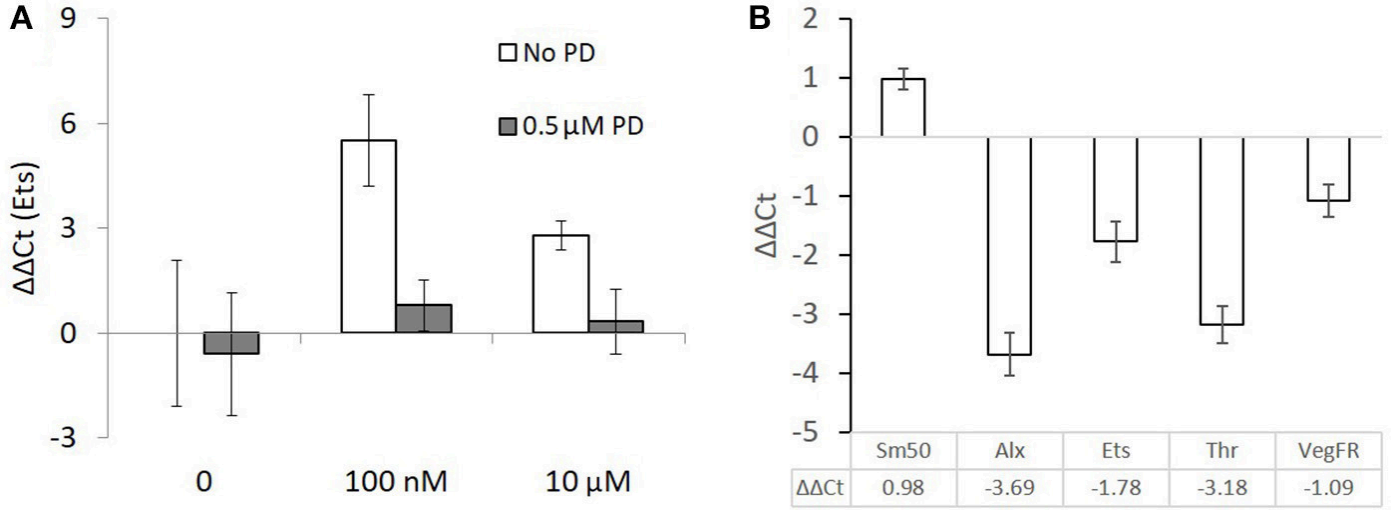
Ets1 upregulation after T4 exposure is prevented by the presence of a MAPK (ERK1/2) inhibitor but skeletogenesis related genes are mostly downregulated in late stage larvae. **(A)** 90 min T4 exposure causes upregulation of Ets1, a regulator of skeletogenesis. This effect is blocked by PD98059, an inhibitor of MAPK. Each group is a single pooled sample of an estimated 1,000–2,000 gastrulae (24 hpf). Mean ΔΔCt with comparison to ubiquitin as a reference gene is displayed with standard error of technical replicates and unexposed larvae from the same culture as a control. **(B)** Larvae were exposed to 100 nM T4 for 24 h before being sampled. These data are taken from 8 pooled samples of 100 larvae each and are relative to an unexposed control group. Mean ΔΔCt with comparison to ubiquitin as a reference gene is displayed with standard error of technical replicates and unexposed larvae from the same culture as a control. Sm50, a marker of skeletogenic activity, is upregulated, while genes normally responsible for regulating skeletogenesis, Alx, and Ets, are downregulated. The genomic thyroid hormone receptor and VEGF receptor are also downregulated. Expression levels were calculated with reference to non-exposed controls.

A pooled sample of 1,000–2,000 early gastrulae (24 hpf), which were incubated in T4 (100 nM or 10 μM) for 90 min showed a large upregulation of Ets1, a transcription factor regulating skeletogenesis which is known to be phosphorylated and activated by MAPK (12). Gastrulae which were pre-incubated with PD98059 before being incubated with T4 did not show an upregulation of Ets1 (Figure 8A). No statistics were performed as a large number of individuals were pooled in a single RNA sample. Both levels of T4 in the absence of PD98059 increased expression levels of Ets1 by 46-fold or 6.9-fold respectively. This effect disappeared in the presence of PD98059.

### PCR of skeletogenic regulatory genes shows downregulation in response to T4 in late-stage larvae

Eight pooled samples of 100 late-stage larvae (24 hpf) were incubated in 100 nM T4 for 24 h. In contrast to the 90-min gastrulae exposure, downregulation of Ets, Alx, and VegfR, genes essential for activating the skeletogenic cell fate in mesenchyme cells and triggering skeletogenesis was observed (Figure 8B). As well, the nuclear thyroid hormone receptor ortholog Thr was downregulated. Still, Sm50, a gene involved in skeletal elongation was upregulated in response to T4 exposure.

### Fluorescently labeled T4 shows T4 binding to PMC membranes

**Figure 9:**
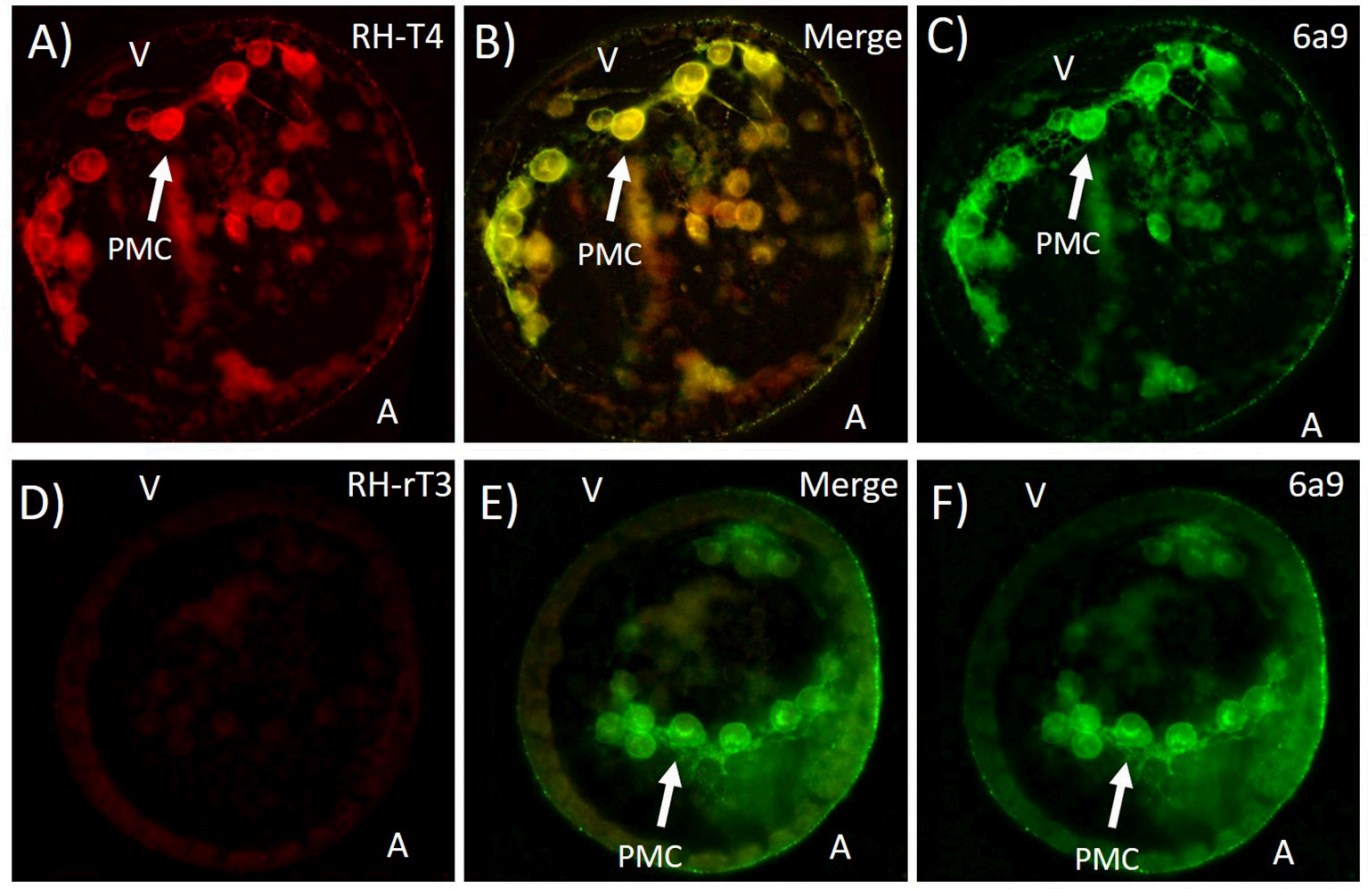
Colocalization of fluorescently labeled T4 (RH-T4—**A**), labeled rT3 (RH-rT3—**D**) with primary mesenchyme cells (PMCs). **(B,E)** Merged image showing colocalization of fluorescently labeled thyroid hormones and 6a9 antibody for PMCs. Gastrulae were incubated with RH-T4 and RH-rT3 for 30 min prior to fixation in methanol. Following fixation, immunohistochemistry with 6a9 antibody **(C,F)** was used to stain the membrane of PMCs. RH-T4 binds specifically to the membrane of PMCs, while RH-rT3 does not, suggesting a specific binding site for T4 in the PMCs.

Rhodamine conjugated T4 bound to primary mesenchyme cells, while RH-rT3 showed no specific binding. Specifically, RH-T4 bound to the membrane and/or extracellular matrix of PMCs. Immunohistochemistry with 6a9, a sea urchin (*S. purpuratus*) specific antibody for the membrane of primary mesenchyme cells was used to confirm binding locations (Figure 9). RH-T4 colocalized with 6a9 staining, suggesting that RH-T4 does bind to the membrane of primary mesenchyme cells. RH-rT3, the biologically inactive control, did not bind to any specific cells, or to specific regions within cells. It also did not colocalize with 6a9 antibody staining to any degree.

### Effects of RGD on T4 activation of MAPK and promotion of skeletogenesis

**Figure 10:**
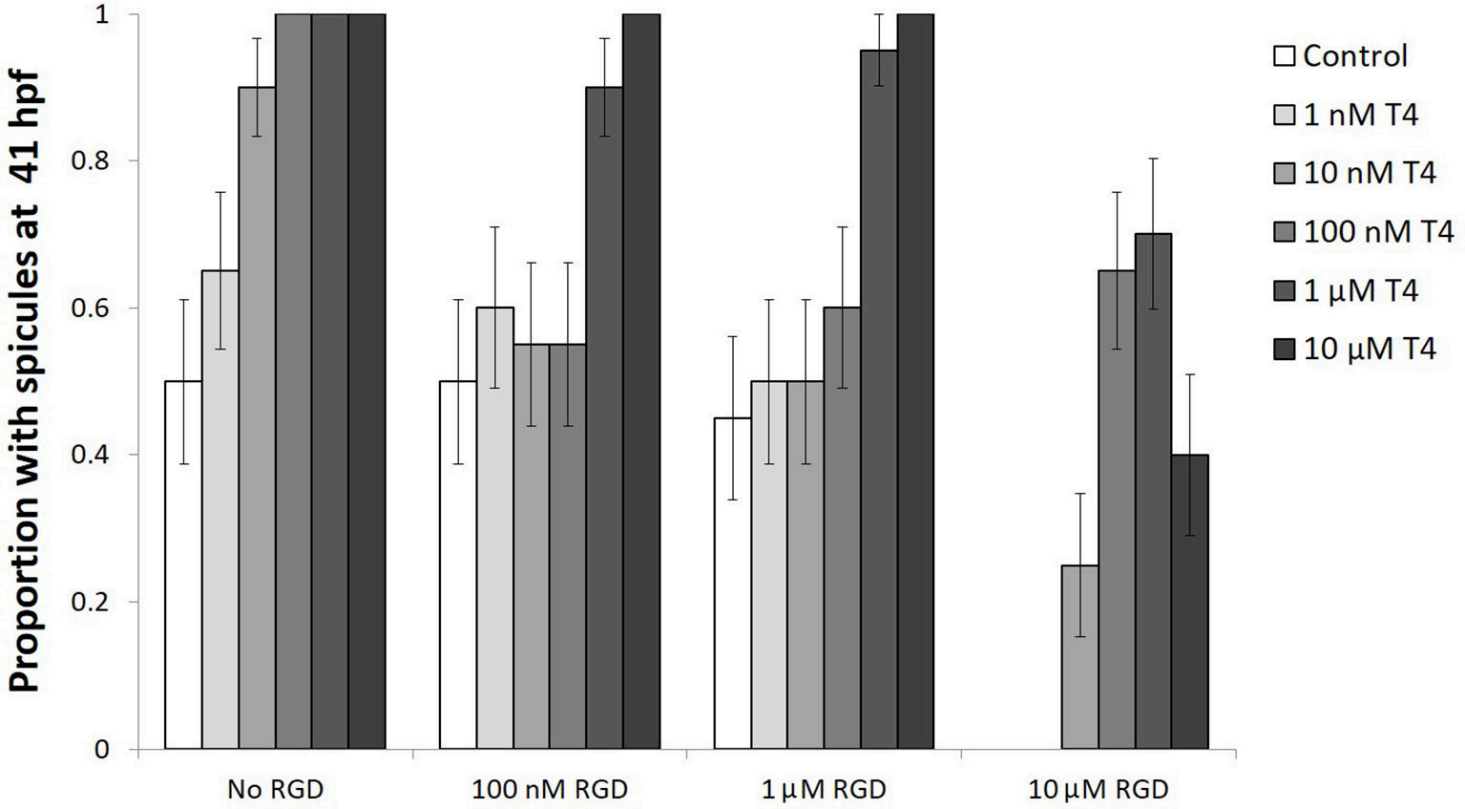
The effect of T4 on skeletogenesis is inhibited by RGD, a small signaling peptide and inhibitor of T4 binding to integrins. Gastrulae were pre-exposed to RGD before being exposed to T4 for 20 h (*n* = 20). Following exposure, spicule proportion at 41 hpf was observed. The hypothesis that RGD inhibits the effect of T4 on skeletogenesis was tested using a binary logistic regression (*D* = 49, *df* = 15). T4 increased the rate of skeletogenesis, while RGD inhibited the effect of T4 on skeletogenesis (Bonferroni corrected *p* < 0.05). The highest levels of RGD prevented skeletogenesis completely, an effect which was rescued by T4 (Bonferroni corrected *p* < 0.05). A significant interaction was detected between RGD and T4, suggesting T4 may bind to an integrin membrane receptor.

Gastrulae were either exposed to T4, RGD, or T4 and RGD and showed increased levels of phosphorylated MAPK and skeletogenesis in the presence of T4, an effect which was inhibited with the addition of RGD (Figure 10). RGD is an inhibitor of T4 binding to integrin αVβ3 in vertebrates which competitively inhibits the binding pocket.

Gastrulae exposed to T4 showed a dose-dependent acceleration of skeletogenesis, having more skeletal spicules after a 20-h exposure (binary logistic regression, Wn = 41.6, *p* < 0.001, Figure 10). The addition of low levels of RGD (100 nM−1 μM RGD) inhibited the effect of T4 on skeletogenesis, except in the presence of the highest levels of T4 tested (1–10 μM T4; Interaction between T4 and RGD, Wn = 2307.42, *p* < 0.001; comparison between control and RGD inhibited groups, *p* = 1.00). Comparison between control and T4 rescued groups, *p* < 0.01). The addition of higher levels of RGD (10 μM RGD) was sufficient to suppress skeletogenesis in all gastrulae that were checked. Skeletogenesis was restored even in the presence of high levels of RGD by addition of T4 (10 nM−10 μM T4; RGD inhibition of skeletogenesis: Wn = 31.8, *p* < 0.001; T4 rescue of skeletogenesis: Wn = 41.6, *p* < 0.001).

**Figure 11:**
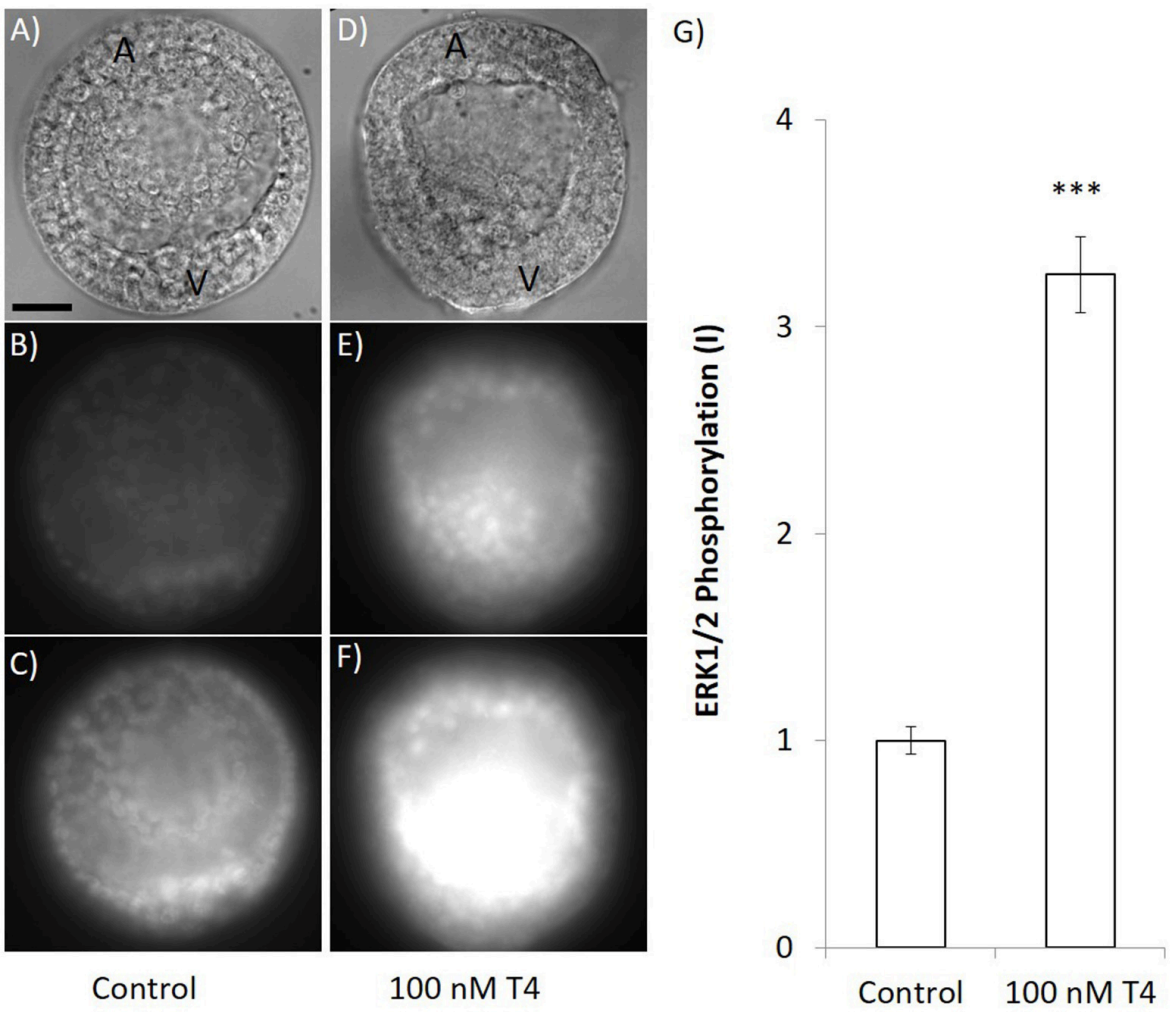
T4 exposure causes phosphorylation of MAPK. Gastrulae were exposed to T4 for 90 min prior to fixation and staining with an antibody that detects the phosphorylated form of ERK1/2 in sea urchins. Panels **(A–C)** show immunohistochemical results when embryos were exposed to T4 carrier control. Panels **(D–F)** show representative images that were taken at the same time and exposure when embryos were treated with 100 nM T4 (**A,D**: DIC image, **B,E**: 133 ms exposure MAPK detection, **C,F**: 400 ms exposure MAPK detection). **(G)** Quantification of the signal revealed that T4 (100 nM) exposed samples fluoresced with significantly higher intensity than the control (3.25 × intensity after subtracting background, 400 ms exposure; ANOVA *n* = 4, ^***^p < 0.001). All images are same scale–scale bar 20 μm. A, animal pole; V, vegetal pole.

We also assessed phosphorylation of MAPK (ERK1/2) after exposure to T4 and found that T4 caused phosphorylation of ERK1/2 (Figure 11). In the group that was not exposed to T4, MAPK was phosphorylated at the vegetal pole of the gastrula, the region in which primary mesenchyme cells form as well as in cells presumed to be PMCs (Figures 11A–C). T4 increased MAPK (ERK1/2) phosphorylation, especially in the vegetal region and presumptive primary mesenchyme cells [*n* = 4; ANOVA; *F*_(1, 6)_ = 132.7, *p* < 0.001; Figures 11D–G].

## Discussion

Thyroid hormones, specifically T4, T3, and T2 had an acceleratory effect on skeletogenesis as measured by skeletal spicule production in early and late stages of development. The effectiveness of an acute exposure, as well as the rapid phosphorylation of MAPK suggests a non-genomic mechanism of action. Inhibitors of both integrin αVβ3 and MAPK (ERK1/2) prevented the acceleratory effect of T4 on skeletogenesis. From these results we hypothesize that T4 signal is mediated via integrin, triggering a MAPK (ERK1/2) signal cascade that ultimately accelerates skeletogenesis.

### Thyroid hormones accelerate skeletogenesis in larval and embryonic sea urchins

In most sea urchins, the larval skeleton begins forming during embryogenesis. The long skeletal arms provide protection from predators and structural support for the ciliated band used in feeding. During early stages, skeleton is deposited by primary mesenchyme cells (PMCs). In *S. purpuratus*, at the 16 cell stage in the early embryo, the micromeres begin differentiating into PMCs. These PMCs then migrate to the blastocoel where they arrange into a ring (23). The location of this ring is coordinated by a signaling protein released by the ectoderm; vascular endothelial growth factor (VEGF) (24). Each PMC cluster initiates skeletogenesis, in the form of a tri-radiate spicule, triggered by a combination of VEGF and MAPK signaling (25).

To test the effects of THs on initiation of skeletogenesis, we developed the assays for early skeletogenesis described here. Since we hypothesize TH affects primarily the initial regulatory controls of skeletogenesis via MAPK, an assay for initiation of skeletogenesis is more informative than assaying elongation, branching, or other skeletal growth. In combination with the skeletal staging scheme developed by Heyland and Hodin (21), it is possible to quantify effects of THs, or other regulatory systems, on initiation of skeletogenesis during every larval stage in which skeleton is produced.

We found T4 to cause phosphorylation of MAPK (ERK1/2) in presumptive PMCs, suggesting that the effect of T4 on skeletogenesis may be attributed to T4 triggering the MAPK cascade that has previously been found to be necessary for skeletogenesis to occur. As well, inhibitors of T4 binding to the receptor reduced apparent phosphorylation, providing clear evidence that T4 stimulates a MAPK cascade.

High levels of T4 alone were sufficient to induce PMCs to form ectopic skeleton during later larval stages. This is consistent with the standard model of sea urchin skeletogenesis in which MAPK signaling is essential for initiation of skeletogenesis. However, it has previously been shown that VEGF signaling is necessary for skeletogenesis (25). A specific VEGF signal is unlikely in the cases of abnormal skeletal growth we described, as they were not isolated to any particular region of the larva. It is possible that under physiologically not relevant doses of T4, a strong MAPK signal is sufficient to initiate skeletogenesis in PMCs which are not in the correct location, explaining the ectopic initiation.

Skeletogenesis in the embryo and larvae can be contrasted with skeletogenesis in the juvenile rudiment. The adult skeleton begins forming in the juvenile rudiment, growing within the larva before metamorphosis (21, 26). The mechanisms of skeletogenesis in the juvenile rudiment are less well-understood, but the morphology has been characterized, and a staging scheme developed in Heyland and Hodin (21). Skeletogenesis in the rudiment is driven by secondary mesenchyme cells, which undergo a transition to become skeletogenic late mesenchymal cells. These cells are similar to primary mesenchyme cells (27). While the signals causing the secondary mesenchymal cells to differentiate into skeletogenic late mesenchymal cells are different than those which induce primary mesenchyme cells, the regulatory gene network governing skeletogenesis in the rudiment is extremely similar (6). As in early larval skeletogenesis, the late mesenchymal cells first form tri-radiate spicules. These spicules subsequently branch out to form either skeletal rings that will support the juvenile tube feet, or spine primordia from which the juvenile spines will extend.

We found T4 to accelerate rudiment development, as has been shown previously in other echinoids (13–15). More specifically, we found that T4 accelerates early skeletal stages in the juvenile rudiment but has no noticeable effect on timing of early soft tissue development. The fact that early soft tissue was not accelerated by thyroid hormones provides evidence that the acceleration of rudiment development found in previous work may be attributed to an effect of T4 on skeletogenesis. This T4 induced acceleration is likely due to a MAPK cascade induced in the skeletogenic late mesenchyme cells found in the rudiment.

Evidence from our qPCR experiments, however, showed conflicting results with respect to a simple upregulation of skeletogenesis in response to TH treatment. Although Sm50, a skeletal protein and marker for skeletogenesis was upregulated by T4 exposure of late stage pluteus larvae, most genes regulating skeletogenesis were downregulated at this stage. Previous work has shown that sea urchins retract their larval arms in preparation for metamorphosis—a developmental event which is accelerated by thyroid hormones (13, 14). The simultaneous acceleration of rudiment growth and larval arm retraction by thyroid hormones may indicate a shift in resources away from larval development toward preparing for metamorphosis. Unpublished work from our lab shows that TH exposure of larvae results in programmed cell death in the arm tips (Wynnen et al. in prep.) and we speculate that this may involve the inhibition or removal of primary mesenchyme cells responsible for growth and maintenance of the larval arms, explaining the seemingly contradictory increase in skeletal activity and decrease in the expression of genes promoting skeletogenesis. In this case, it could be attributed to a difference in the effect of thyroid hormones on the primary mesenchyme cells in the larval arms and the late mesenchyme cells in the rudiment. This difference would seemingly only manifest later in development, given the capability of thyroid hormones in early development to stimulate PMC initiation of skeletogenesis.

Thyroid hormones have been shown to be both apoptotic and anti-apoptotic through different pathways in vertebrates. For example, when acting via integrin-mediated MAPK, T4 was able to prevent p53-dependant apoptosis in human cell lines (28). During amphibian metamorphosis, however, T3 acting via the nuclear thyroid hormone receptor causes extraordinary apoptosis required for tissue remodeling [reviewed in (29)]. It is possible that the differential effects of thyroid hormones in sea urchins stem from a differing effect of genomic and non-genomic pathways on apoptosis and cell proliferation.

### T4 signals through a putative non-genomic pathway in sea urchin embryos

**Figure 12:**
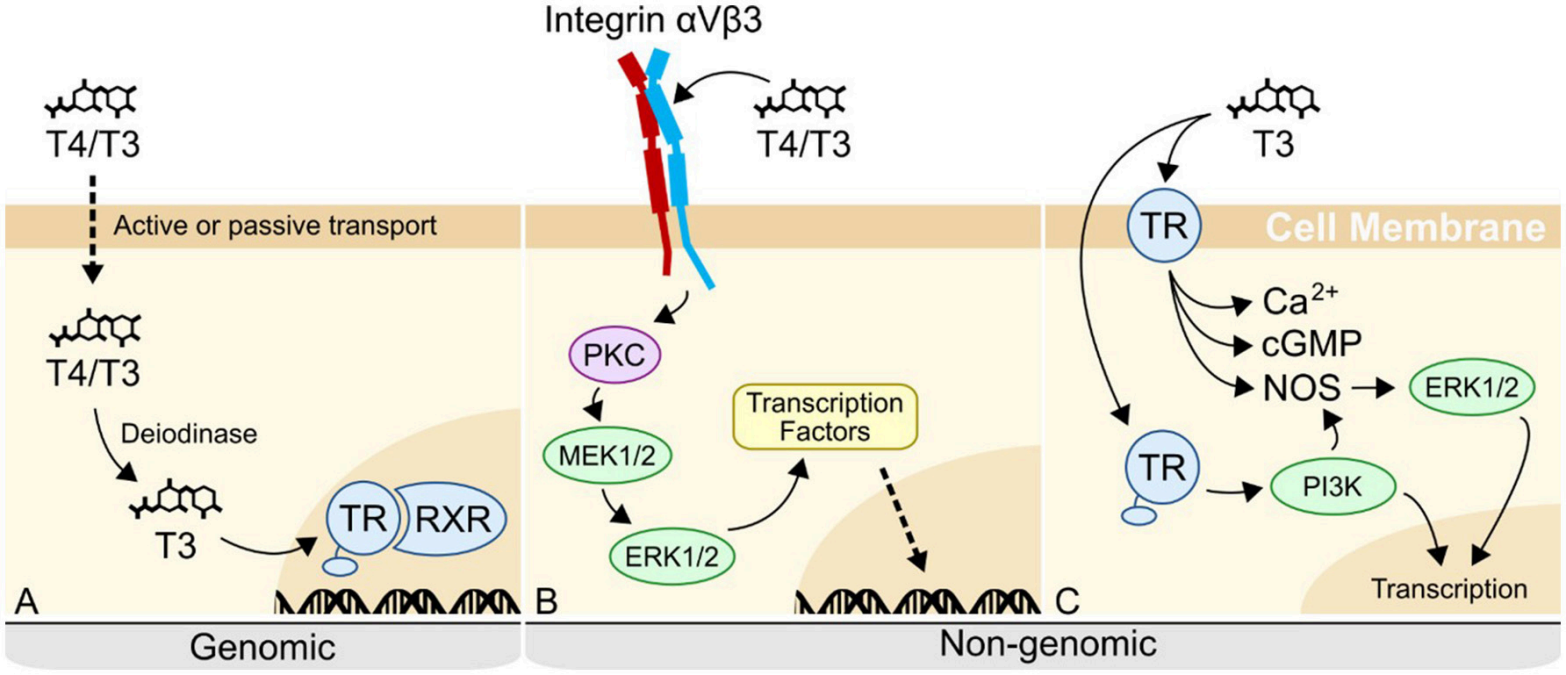
Genomic and non-genomic mechanisms of thyroid hormone action in vertebrates. **(A)** In the canonical genomic signaling, T4 is released by the thyroid gland, before being deiodinated into T3. T3 then binds to the nuclear thyroid hormone receptor (TR) in complex with the retinoid X receptor (RXR) at hormone response elements in the genome, regulating transcription. **(B)** In non-genomic signaling, T4/T3 can bind to an integrin membrane receptor. When binding to integrin αVβ3, T4/T3 triggers a MAPK cascade, resulting in the phosphorylation and activation of transcription factors. **(C)** Extranuclear TR is the other described receptor for non-genomic activity. T3 binds to cytoplasmic or membrane-bound TR, phosphorylating PI3K, as well as triggering Protein Kinase G (PKGII) or nitric oxide (NO) signaling pathways through the activation of cGMP and NO Synthase (NOS) respectively. See (30) for review of currently known non-genomic pathways.

What then, is the mechanism by which THs regulate sea urchin skeletogenesis and the mesenchymal cells responsible? In vertebrates, THs regulate mesenchymal cells via both genomic and non-genomic pathways (Figure 12). Genomic mechanisms involve the nuclear thyroid hormone receptor (TR) binding to thyroid hormone response elements (TRE) within the promoter region of target genes [reviewed in (31)]. Many functions have been attributed to genomic actions of thyroid hormones, including regulation of metabolism, differentiation, and apoptosis. For example, frog metamorphosis is famously regulated by the action of the nuclear thyroid hormone receptors (32). In contrast to the genomic mechanism, non-genomic mechanisms of thyroid hormone action are diverse and less well-studied in comparison to genomic mechanisms [reviewed in (30)].

Generally, non-genomic mechanisms are characterized by a more rapid response time, owing to the phosphorylation and activation of proteins. Research on non-genomic mechanisms has so far focused on two possible receptors: extranuclear TR, and the membrane receptor, integrin αVβ3. Most vertebrate cell types are capable of responding to T4 via integrin-mediated signaling (33). T4 binds to a site within the RGD binding pocket of integrin αVβ3, triggering a mitogen-activated protein kinase (MAPK) signaling cascade through ERK 1/2. This process has been shown to induce angiogenesis (30, 34), regulate sodium channels during development of the nervous system [reviewed in (35, 36)] and induce proliferation of osteoblasts (37), among other functions [reviewed in (30)]. In contrast to vertebrates, invertebrate mechanisms are largely unknown, despite an abundance of information in the literature that THs affect the physiology and development of invertebrate species, such as sea urchins, molluscs, and cnidarians [(13, 14, 16, 38–40), reviewed in (41, 42)].

There have been previous efforts to characterize TH binding to a putative nuclear receptor in sea urchins (V. Laudet, pers. com.). Similarly, attempts to characterize genomic mechanisms in other invertebrates have been so-far unsuccessful [reviewed in (42)]. In the oyster, *Crassostrea gigas*, a TR ortholog was cloned and found to bind both THs and hormone response elements (39). However, T4, T3, and Triac did not cause the TR ortholog to promote transcription, as determined by a luciferase assay. It is possible that some TH actions in invertebrates, as in vertebrates, are mediated by non-genomic mechanisms.

Some effects observed in *S. purpuratus*, namely initiation of skeletogenesis in gastrulae, occur and are affected by TH exposure on shorter time-scales than would be expected for a nuclear receptor. Non-genomic mechanisms of TH action are often characterized by their rapid response times. For example, MAPK cascades achieve peak phosphorylation in 3–15 min (43) as compared to TH binding to the nuclear thyroid hormone receptor which often requires hours to days to have a regulatory effect [e.g., during amphibian metamorphosis, see (32) for review]. In sea urchin gastrulae, a 20-h exposure to THs drastically accelerates skeletogenesis. As well, acute exposure of only 1 h to high levels of T4 is sufficient to cause acceleration of skeletogenesis in 12 day-old larvae. Finally, Ets1 expression level changes are observed in gastrulae after only 1–1.5 h exposures. This suggests that a non-genomic mechanism may mediate TH actions on skeletogenesis in sea urchin larvae.

In vertebrates, a combination of VEGF and TH-mediated MAPK (ERK1/2) signaling regulates angiogenesis through phosphorylation of Ets proteins (44, 45). Sea urchin skeletogenesis also requires VEGF and phosphorylation of Ets1, a regulatory module which was presumably only activated in the adult urchin ancestrally, but evolutionarily became co-opted to regulate larval skeletogenesis as well (46).

**Figure 13:**
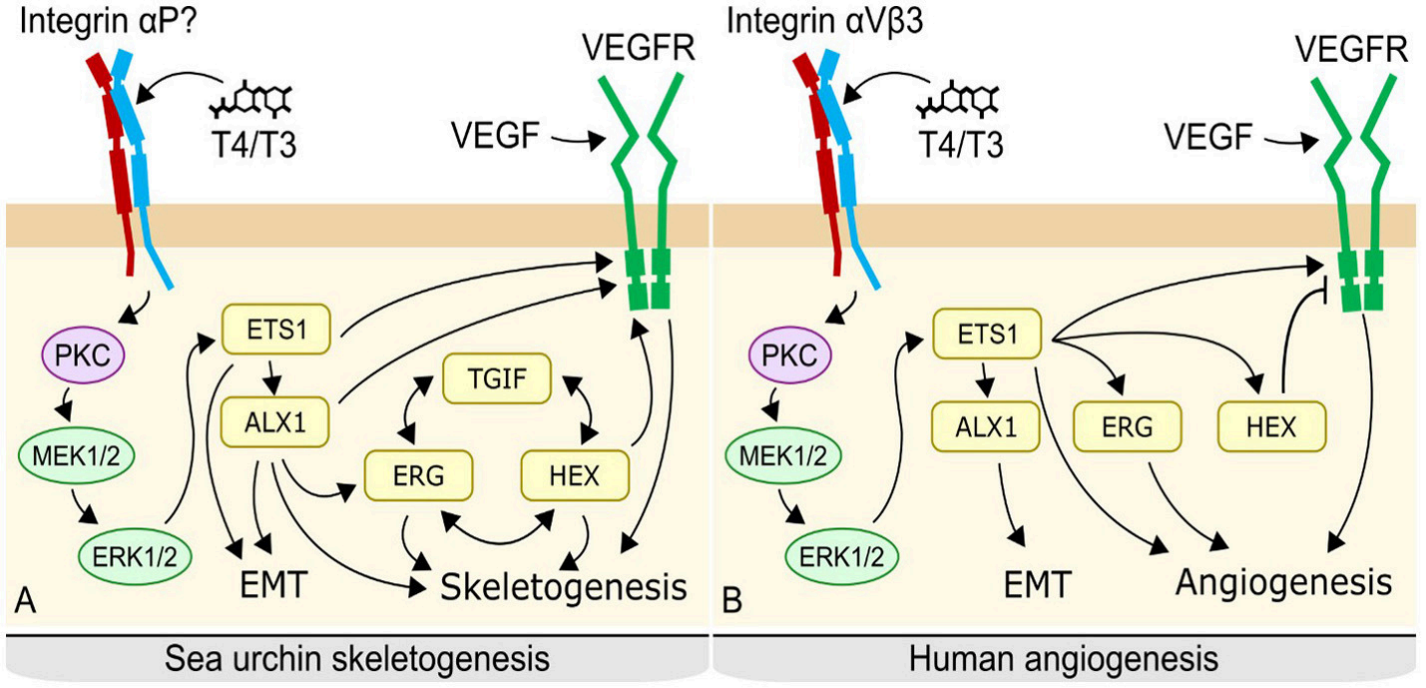
Proposed mechanism of thyroid hormone action in sea urchins **(A)** and potentially conserved system in vertebrates. **(B)** In sea urchins, thyroid hormones likely bind to an integrin on the surface of primary mesenchyme cells, triggering a MAPK cascade. MAPK phosphorylates Ets1 and Alx1, activating them. Ets1 and Alx 1 upregulate regulatory controls of skeletogenesis, leading to initiation of skeletogenesis as well as ingression of the primary mesenchyme cells; an epithelial to mesenchyme transition. A Tgif-Hex-Etg regulatory loop maintains the skeletogenenic fate of the cell and also promotes skeletogenesis. In vertebrates, thyroid hormones bind to integrin αVβ3 on the membrane of fibroblasts, triggering a MAPK cascade. MAPK phosphorylates Ets, promoting angiogenesis, and epithelial to mesenchyme transition via Alx. Some details differ, notably the lack of direct regulation of angiogenesis by Alx in the vertebrate mechanism, the absence of the Tgi-Hex-Erg regulatory loop, and the inhibition of VegfR by Hex.

There are differences between vertebrate and sea urchin mesenchymal regulation, notably the lack of certain transcription factors such as Alx1 in vertebrates, as well as the presence of a negative feedback loop regulating the VEGF receptor, VEGFR. While the function of some transcription factors differs between the two systems, evidence suggests these systems may be conserved modules of mesenchymal cell regulation which have evolved divergent functions in sea urchins and vertebrates (Figure 13).

One commonality, however, is the increased efficacy of T4 over T3. In vertebrate non-genomic thyroid hormone signaling, T4 is preferentially bound by integrin αVβ3. In all experiments where multiple thyroid hormones were tested in sea urchins, T4 resulted in the strongest effect on skeletogenesis, in both early and late stage embryos and larvae. In contrast to T4, T3 and T2 had a weaker effect on skeletogenesis but were overall similar to each other. Together with our finding that these TH hormones showed a similar magnitude of acceleration of skeletogenesis, but at higher concentrations, this suggests that these hormones have a lower binding affinity to their putative receptors. Triac had an inhibitory effect on skeletogenesis, while Tetrac and rT3 show no effect on the rate of initiation of skeletogenesis. These results are consistent with integrin-mediated TH signaling in vertebrates, where T4 has a higher binding affinity than T3, and Triac acts antagonistically to T4. This is also consistent with how TH signaling has been shown (so far) to work in other invertebrates. In all cases discovered, T4 is the most active TH [reviewed in (42)].

In gastrulae, T4 was found to bind to the membrane, and/or the extracellular matrix of primary mesenchyme cells. PMCs are the cells which build skeleton in larval sea urchins. The colocalization with 6a9, an antibody for the membrane of PMCs, was complete, varying only in intensity but not localization. The binding of RH-rT3, a rhodamine conjugated form of the non-biologically active form of T3, was entirely non-specific. The lack of specific binding of RH-rT3 suggests that the specific binding of RH-T4 reflects actual binding locations of T4. The T4 receptor might therefore be found in the PMC membrane or extracellular matrix. We cannot exclude a nuclear receptor with these results, as it is possible that RH-T4 would not make it through the membrane and into the nucleus or cytoplasm. It is also possible that RH-T4 bound to a thyroid hormone transporter, and not to a functional receptor, though we believe this to be unlikely considering the evidence for an integrin mediated mechanism.

High levels of T4 can cause skeletal abnormalities such as protrusions from the larval arms, duplicate larval arms, or ectopic skeleton. T3 was only found to stimulate the growth of duplicate arms, but not the formation of ectopic skeleton or protrusions. Activation of skeletogenesis in migrating PMC cells, or cells not at usual sites of skeletogenesis, suggests that high levels of T4 may be sufficient to initiate skeletogenesis without VEGF or other external signals. This only occurs with very high levels of T4, however, and it is likely that T4 is not normally the sole initiator of skeletogenesis in sea urchin larvae. Previous work has shown similar effects of MAPK and VEGF on gene expression levels (25), proposing that VEGF acts through MAPK. Should both T4 and VEGF signaling be MAPK-mediated, it may explain the ability of high levels of T4 to bypass the usual requirement for VEGF to initiate skeletogenesis.

In vertebrate mesenchyme cells involved in angiogenesis, T4 binds to integrin αVβ3. A potential homolog of the integrin αV subunit has been identified (47), that is expressed throughout sea urchin larval development, including during gastrulation and later larval stages. In vertebrates, T4 binds to the same binding pocket as RGD peptide on the integrin αV subunit. When tested in *S. purpuratus* larvae, RGD had an antagonistic relationship with T4 activity.

RGD peptide prevented or inhibited skeletogenesis, depending on the concentration used. Skeletogenesis was rescued by higher levels of T4, but not by lower levels. The data are consistent with a competitive inhibition similar to that shown in vertebrates (34). It is still possible that RGD, being a signaling peptide, has other effects on skeletogenesis. However, subsequent tests with immunohistochemistry of phosphorylated MAPK suggest otherwise.

In the control group, cells in the blastocoel at the vegetal pole of the gastrulae, presumed to be primary mesenchyme cells, express phosphorylated MAPK (ERK1/2). This indicates that ERK is activated in PMCs during normal gastrulation and has been seen previously (48). However, addition of T4 greatly increases the quantity of phosphorylated MAPK at the vegetal pole, as well as causing some MAPK phosphorylation in the basal membranes of all ectodermal cells. The induction of phosphorylated MAPK in non-PMC cells suggests that T4 may have effects in the developing embryo which are not limited to skeletogenesis. This is further preliminary evidence that T4 may bind to an integrin membrane receptor in sea urchins, a hypothesis that needs to be tested in more details in future studies.

Two MAPKs have been found to be relevant to skeletogenesis in sea urchins, ERK1/2, and p38. Of those, ERK1/2 is necessary for skeletogenesis to occur and is known to act by phosphorylating Ets1, a transcription factor regulating skeletogenesis (48). While SB203580, an inhibitor of p38, did not prevent the effect of T4 on skeletogenesis, PD98059, an inhibitor of ERK1/2, did prevent the acceleration of skeletogenesis by levels of T4 under 10^−6^ M. These data suggest that ERK1/2 is a necessary intermediary for the skeletogenesis caused by T4 at physiologically relevant concentrations of T4. Interestingly, the highest levels of PD98059 tested prevented skeletogenesis entirely, an effect which was partially rescued by T4. This confirms that MAPK (ERK1/2) signaling is essential for skeletogenesis in sea urchins, as was previously found by Röttinger et al. (12).

ERK 1/2 phosphorylates Ets1, thereby regulating a variety of genes which are differentially expressed in primary mesenchyme cells (11). qRT-PCR of Ets1 after a short 90-min exposure to T4, showed Ets1 to be greatly upregulated. This effect vanished in the presence of PD98059, meaning the effect of T4 on the upregulation of Ets1 is also MAPK (ERK1/2) mediated. This is consistent with results from (48) showing downregulation of Ets1 in the presence of MAPK inhibitors.

The increase in Ets1 expression after T4 exposure suggests that the phosphorylation of Ets1 and its subsequent activation leads to the autoinduction of Ets1, in a positive feedback loop. Further, it implies regulation by TH of the transcription factors regulating initiation of skeletogenesis. This is reinforced by morphological results of T4, T3, and T2 in gastrulae, and 12 day-old larvae, where we found an acceleration of initial spicule formation. Thyroid hormones, mainly T4, likely trigger initiation of skeletogenesis by binding to an integrin on mesenchymal cells, activating a MAPK cascade phosphorylating Ets1.

### Evolutionary implications of non-genomic TH signaling in sea urchins

The prevalence of T4 as the primary TH signaling molecule in sea urchins as well as the evidence for non-genomic TH signaling presented here has important implications for the origins and evolution of TH signaling in animals. Thyroid hormones likely preceded the evolution of animals as they can be commonly found in plants, protists and bacteria (18, 49). It is therefore likely that receptor signaling systems in animals evolved in response to the presence of these hormones. This hypothesis is in part supported by the fact that many invertebrates respond to TH in some fashion, however the mechanisms and functions of these responses are poorly understood [for review see (41, 42)]. Still, recent experimental data has uncovered new evidence for TH signaling mechanisms and regulatory functions in molluscs, sea urchins, ascidians, and amphioxus [for example, (14, 39, 50–52)].

A genomic mechanism of TH action in invertebrates has not yet been conclusively shown. One possibility is that the thyroid hormone which activates these receptors is undiscovered and is a perhaps a metabolite such as T2. T2 is known to differentially activate TR paralogs in teleosts (53), and has not yet been tested with non-chordate TR. Another possibility is that in molluscs and potentially other invertebrates including echinoderms, TR is an orphan receptor or binds another ligand, and that TH signaling proceeds mainly via non-genomic mechanisms.

Some aspects of thyroid hormone action once attributed to genomic action are now being suggested to be non-genomic in nature (31). We propose that non-genomic actions of thyroid hormones are under-studied. A bias toward the historically first discovered mechanism of thyroid hormone action, the canonical genomic pathway, has dominated investigations into thyroid hormone action in non-chordates especially. It seems increasingly likely that non-genomic mechanisms may be responsible for the regulation of developmental processes by TH which has been observed in some invertebrates. For example, skeletogenesis or metamorphosis have been reported to be regulated by T4 in various Cnidaria, including Aurelia, Cassiopea, and some corals (40, 54, 55). T4 is the only thyroid hormone present in Cnidaria (56), and is the most active hormone in sea urchins and molluscs. Notably, Cnidaria do not possess a nuclear thyroid hormone receptor ortholog (56), and any regulatory mechanisms of T4 could be non-genomic or using alternative nuclear hormone receptors. Having now shown a T4 stimulated non-genomic mechanism regulating skeletogenesis in urchins, this model of non-genomic signaling in invertebrates is supported by some preliminary evidence.

## Author contributions

All authors listed have made a substantial, direct and intellectual contribution to the work, and approved it for publication.

### Conflict of interest statement

The authors declare that the research was conducted in the absence of any commercial or financial relationships that could be construed as a potential conflict of interest.
